# Barriers to mental health care among severe mental illness in rural India: A qualitative study

**DOI:** 10.6026/973206300213260

**Published:** 2025-09-30

**Authors:** Thakur Shakti Singh, Rajkiran Salunkhe, Murali M.R., Heena Dixit, Rahul Tiwari, Nirvi Sharma, Akriti Mahajan

**Affiliations:** 1 Department of Psychiatry, SVS Medical College, Mahabubnagar, Hyderabad, Telangana, India; 2 Department of Psychiatry, Government Medical College, Miraj, Maharashtra, India; 3 Department of Psychiatry, JSS Academy of Higher Education and Research, Mysuru, Karnataka, India; 4 Department of Medical Health Administration, Index Institute, Malwanchal University, Indore, Madhya Pradesh, India; 5 Department of Oral and Maxillofacial Surgery, RKDF Dental College and Research Centre, Sarvepalli Radhakrishnan University, Bhopal, Madhya Pradesh, India; 6 Department of Pediatric Occupational Therapy, Jaipur Occupational Therapy College, Jaipur, Rajasthan, India; 7 Department of Oral Medicine and Radiology, Private Consultant, Jammu and Kashmir, India

**Keywords:** Rural health, mental health services accessibility, severe mental illness, qualitative research, India

## Abstract

Individuals with severe mental illness and their caregivers face multifaceted barriers to accessing mental health care in rural India. Semi-structured interviews with 20 participants revealed key impediments including travel distance, financial burden, limited service availability, stigma, reliance on traditional healers and poor illness insight. Thus, we show the need for culturally tailored, community-based and telepsychiatry solutions. Strengthening primary care through integration and caregiver support may enhance access and engagement.

## Background:

India exhibits a substantial rural-urban disparity in mental health care access, with rural areas bearing a disproportionate share of unmet needs [1]. The National Mental Health Survey (2015-16) reported treatment gaps ranging between 70% and 92% across different disorders, underscoring clear systemic deficiencies [2]. Structural limitations include inadequate infrastructure, limited psychiatric workforce and sparse availability of inpatient facilities in rural settings [3]. This is compounded by the workforce gap, with only 0.75 psychiatrists per 100,000 populations, far below WHO recommendations of 1.7 and distribution skewed toward urban areas [4]. In rural India, poverty and lack of public transport exacerbate obstacles, making proximity to care centers a critical barrier [5]. Cultural factors such as pervasive stigma and reliance on faith healers deter many from seeking formal care, delaying treatment initiation [6]. Mental health literacy in rural populations remains low, with misunderstandings about illness etiology and treatment contributing to reluctance in help-seeking [7]. Furthermore, limitations in primary care integration and low availability of trained personnel hinder early detection and referral [8]. Although digital interventions show potential to bridge geographical gaps, adoption remains nascent in rural contexts due to infrastructural and literacy challenges [9]. Qualitative exploration of lived experiences is essential to understand nuanced, context-specific barriers that quantitative surveys may overlook [10]. Therefore, it is of interest to study of barriers to mental health care among severe mental illness in rural India.

## Materials and Methods:

A descriptive qualitative study was conducted from February to May 2025 in two rural areas. Participants included adults (≥18 years) diagnosed with severe mental illness-namely schizophrenia, bipolar disorder, or major depressive disorder with psychotic features-and their primary caregivers. Using purposive sampling through community mental health outreach programs, we recruited 20 participants: 10 patients and 10 caregivers. Semi-structured, in-depth interviews (~45-60 minutes) were conducted in local languages (Telugu or Hindi), in participants' homes or at nearby health centers, following a standardized interview guide. Topics included help-seeking behavior, perceived obstacles to care, illness understanding and suggestions for improving access. Interviews were audio-recorded, transcribed verbatim and translated into English. Two researchers independently performed thematic analysis. Initial coding was followed by iterative grouping into subthemes and overarching themes. Researchers met regularly to resolve discrepancies and refine codebooks; data saturation was achieved by the 18th interview. Demographic and contextual data were captured: patient age, gender, household income and distance to nearest psychiatric facility. Median values are reported. Ethical approval was granted by the appropriate Institutional Ethics Committee. Written informed consent was obtained, with assurance of confidentiality and data anonymization.

## Results:

Table 1 (see PDF) shows that participants were predominantly middle-aged (mean 38.8 years), with males slightly more represented (55%). A large proportion (65%) reported monthly household income below INR 10,000, underscoring financial vulnerability. Additionally, 75% lived more than 30 km from the nearest psychiatric facility, highlighting geographical barriers that compound economic hardship, thereby restricting timely access to care (Table 1 - see PDF). Table 2 (see PDF) highlights multiple barriers to mental health care. Financial and travel costs were reported by 70% of participants, while 75% cited long travel distances. Stigma was a major deterrent (65%), with nearly half turning to faith healers (45%). Poor illness insight was observed in 40% of cases. These overlapping barriers illustrate the structural, societal and illness-related obstacles that reduce engagement with formal services and delay treatment seeking in rural India (Table 2 - see PDF).

## Discussion:

This qualitative investigation elucidates the layered and interconnected barriers hindering access to mental health care for individuals with severe mental illness in rural India. Service-related barriers are foundational. The median 35 km travel distance aligns with previous reports of "mental health deserts" in rural India, where geographic isolation restricts service accessibility [11]. Financial strain was pervasive, as 70% of participants cited combined travel and consultation costs as prohibitive-reflecting the broader context in which India's rural populations bear high out-of-pocket expenditures for health services [12]. The psychiatric workforce shortage is stark; India's 0.75 psychiatrists per 100,000 population falls significantly short of WHO's benchmark, especially as rural allocation remains disproportionately low [13]. This skews service availability toward urban centers and contributes to irregular clinic access in rural zones.

## Societal forces further amplify access deficits:

Stigma-reported by 65% of participants-emerges as a decisive deterrent to formal help-seeking. Such findings are consistent with North-Indian situational analyses documenting stigma's profound effect on treatment uptake and retention [14]. The culturally entrenched use of faith healers, reported by 45% of participants, mirrors national patterns wherein traditional and religious pathways often precede biomedical care [15]. Low mental health literacy compounds these issues, with misunderstandings about mental illness leading to delayed interventions and reluctance to engage with psychiatric treatment [14]. Illness-related barriers, though often underemphasized, substantially influence care dynamics. Poor insight, observed in 40% of patients, commonly leads to treatment refusal-a barrier well-documented in severe mental illnesses [16]. Persistent symptomatology despite treatment further discouraged caregivers and patients, reinforcing discontinuation of help-seeking. Interconnectedness of barriers is noteworthy: geographic and financial hurdles intensify social reluctance; stigma discourages help-seeking, increasing reliance on ineffective traditional care; illness-related challenges manifest more acutely when support systems and accessible services are lacking.

## Targeted interventions are essential:

[1] Telepsychiatry and decentralization of care: Given the geographic barriers, telepsychiatry integrated within primary health centers offers promise. Pilot programs signal improved access and engagement when coupled with telephonic or community-based counseling [17].

[2] Community engagement and anti-stigma campaigns: Community engagement and anti-stigma campaigns are critical, as stigma and cultural beliefs often lead patients to discontinue care. In fact, nearly half of patients in an Indian district-level mental health program dropped out after their first visit, with overall 64.3% discontinuing within a year-underscoring the urgent need for community outreach strategies [18].

[3] Capacity building in primary care: Training ASHA workers and primary care staff to screen, counsel and refer patients could bridge access gaps. The Ayushman Bharat scheme's inclusion of mental health in primary health care frameworks provides a viable infrastructure for this integration [19].

[4] Financial support mechanisms: Subsidizing travel or providing teleconsultation exemptions for low-income households can reduce the financial burden, particularly for those earning under INR 10,000 monthly-prevalent in this study's sample (65%).

[5] Psychoeducation and caregiver support: Enhancing understanding of illness, treatment expectations and symptom management may improve insight and adherence. Community-based psychoeducation interventions have shown efficacy in increasing engagement among patients with severe illnesses and their caregivers [20].

Limitations must be acknowledged. Findings are drawn from two rural districts in Andhra Pradesh and may not generalize across India's heterogeneous regions. Social desirability bias may have influenced participant reporting. Also, the qualitative sample size, though adequate for thematic saturation, limits quantitative inference. Nevertheless, the study's strengths include rich qualitative data, inclusion of both patients and caregivers and the embedding of quantitative descriptors that bridge depth and context. Future research should explore co-designed interventions leveraging telepsychiatry, community-led stigma reduction and primary care integration. Evaluating these through mixed-methods and cluster-randomized designs would inform scalable policy models.

## Conclusion:

Severe mental illness in rural India is hindered by intertwined service, societal and illness related barriers. Integrated culturally tailored strategies-including telepsychiatry, community engagement, financial support and caregiver empowerment are vital to improving access and outcomes.
